# Taking stock of intersectionality-informed policy guidance for post-pandemic recovery: a scoping review

**DOI:** 10.1186/s12939-026-02853-8

**Published:** 2026-05-23

**Authors:** Gemma Hunting, Olena Hankivsky, Ashlee Christoffersen

**Affiliations:** 1 Interdisciplinary Studies Graduate Program, Simon Fraser University, Vancouver, BC Canada; 2 School of Public Policy, Simon Fraser University, Vancouver, BC Canada; 3 Department of Politics, York University, Toronto, ON Canada

**Keywords:** Intersectionality, COVID-19, Public policy, Equity, Policy guidance

## Abstract

**Background:**

COVID-19 was a catalyst for popularizing intersectionality, an approach now recognized as central to understanding the inequitable consequences of COVID-19 and as a necessary lens for recovery. Yet, a key question remains: *Did the pandemic provide the stimulus for improving or advancing operational policy-relevant guidance for intersectionality?* Answering this is critical, given persistent calls for practical policy guidance for operationalizing intersectionality. This article explores this question by first providing a brief overview of intersectionality, including its value-added in relation to equity-promoting public policies. We then summarise how intersectionality was taken up during and post-pandemic, situating these trends within the broader field of intersectionality-informed research and policy. Following this, we present findings derived from a review of guidance for applying intersectionality in the context of COVID-19.

**Methods:**

The literature reviewed derives from a larger scoping review (conducted by the authors) of guidance (including tools, guides, and frameworks) intended to facilitate the application or operationalization of intersectionality as relevant to public policy. Inclusion criteria for the scoping review included English language, any date (to end of 2023), presents user-friendly guidance, mentions policy, and contains intersectional* in the title.

**Results:**

Our findings show that despite much discussion on intersectionality in relation to COVID-19 and the increase in intersectionality guidance since the outbreak, *little guidance was developed on how to conduct intersectionality-informed analysis in relation to COVID-19 or post-pandemic recovery*. Yet, our analysis also shows that the guidance focused on COVID-19 stands out as more robust than the majority of intersectionality guidance produced to date. Specifically, the COVID-19 guidance *reflects the foundational tenets of intersectional analysis and action, boosting its potential to capture and address inequities*. This is in contrast to the broader scoping review which indicates that most existing intersectionality guidance has failed to do this.

**Conclusion:**

It is pressing that researchers, policy actors and practitioners who seek to take up intersectionality in their work critically take stock of promising practices in intersectionality guidance – exemplified by the COVID-19 guidance reviewed in this article - to strengthen how inequities are captured and addressed.

**Supplementary Information:**

The online version contains supplementary material available at 10.1186/s12939-026-02853-8.

## Background

When COVID-19 began, the UN Secretary-General, António Guterres, referred to it as the most challenging crisis since the Second World War. The pandemic was arguably a catalyst for popularizing intersectionality, an approach which had steadily gained traction in the previous 15 years across research, policy and practice spheres as necessary to capture and address the complexities of inequity. Intersectionality is now widely recognized as central to fully understanding the inequitable consequences of COVID-19 [1], [2–13], and as a necessary lens for post-pandemic recovery [14–20]. However, an important question remains: *Did the pandemic provide the stimulus for improving or advancing operational policy-relevant guidance for intersectionality?* Answering this question is critical, given that steady calls persist internationally for clear and context relevant guidance for operationalizing intersectionality across researchers, policy actors, and advocacy groups [3–6, 14, 20–28].

This article focuses on answering this question. We first provide a brief overview of intersectionality, including its key tenets and value-added in relation to equity-promoting public policies. We then summarise how intersectionality was taken up during the pandemic, from the outbreak to recovery efforts, and situate these trends within the broader field of intersectionality-informed research and policy internationally. Following this, we present findings derived from a scoping review of guides, tools or frameworks (herein referred to as guidance) for applying intersectionality in the context of COVID-19. This review focuses particularly on public policy relevant guidance, as it is in this area where calls to take up intersectionality have been steadily increasing while new forms of operational guidance to do this is developing, however, as a whole, such guidance has not been comprehensively evaluated.

Our findings indicate that despite the explosion of research and discussion on intersectionality in relation to COVID-19, including public calls for its deployment to address inequities, *little guidance was developed on how to conduct intersectionality-informed analysis specifically in relation to COVID-19 or post-pandemic recovery*. This is surprising, given that our broader scoping review of all intersectionality guidance to date (including and beyond those focused on the pandemic) identified that the majority of such guidance proliferated from 2020 onwards [29]. This suggests that the pandemic may have propelled the popularity of taking up intersectionality in relation to other issues and sectors beyond COVID-19.

Notably, our analysis shows that the guidance produced on operationalizing intersectionality in relation to COVID-19 - seven in total - stands out as more robust than the majority of intersectionality guidance produced to date. Specifically, the COVID-19 guidance accurately *reflects the foundational tenets and intentions of intersectional analysis and action, boosting its potential to truly capture and address inequities in transformative ways* [29]. This is in direct contrast to our broader review findings which indicate that most existing intersectional guidance, published both before and after the pandemic, fails to do this. Given this disjuncture, it is pressing that researchers, policy actors and practitioners who seek to take up intersectionality in their work critically take stock of promising practices in intersectionality guidance – exemplified by the guidance reviewed in this article - to strengthen how inequities are captured and addressed.

### Intersectionality

Emerging from Black feminist activism and critical race theory, intersectionality is an interdisciplinary framework for analyzing how various factors interact with one another to create inequities and privileges across individuals, groups, processes, and structures [30–33] While definitions vary, intersectionality promotes an understanding of individuals as shaped by the interaction of different social locations (e.g., gender, race, age, Indigeneity, class, sexuality, geography, age, dis/ability, migration status). These interactions occur within a context of connected systems and structures of power (e.g., policies, laws, institutions, media). Through such processes, interdependent systemic bases of privilege and oppression derived from forces including colonialism, racism, homophobia, ableism and patriarchy are created [33].

In short, intersectionality: 1) asks researchers, policy actors and decision makers to critically reflect on how their identities, perspectives, and knowledges influence how they see specific policy problems and populations; 2) considers multiple and interacting processes and systems of inequity and discrimination simultaneously; 3) utilizes multiple levels of analysis, including the biological, interpersonal, institutional, and societal, to engender conceptual shifts in how systems of advantage and disadvantage are understood; 4) captures context specific dimensions of experience related to time and place; 5) engages in methods that privilege the perspective of groups experiencing multiple forms of subordination; and 6) prioritizes a focus on human resilience and social justice.

Intersectionality challenges partial or reductive understandings of and solutions to policy problems by generating knowledge that reflects the complexity of human experiences and needs across the life course. In the absence of an intersectional approach, for example, researchers and policy actors tend to disaggregate inequitable outcomes using discrete categories such as gender, socioeconomic status (SES), and race, highlighting differences between groups (e.g., women vs. men) without capturing within-group differences or making the complex contexts that drive inequities explicit [34]. Even when more than one factor is considered, approaches are often additive (e.g. gender+SES+race), failing to capture inequities and advantages created at the intersection of factors. To move beyond these limitations, intersectionality requires cross-disciplinary and sectoral work to capture the co-constitutive nature of the multi-level factors shaping health and social issues, and identify possible levers of intervention. In its ability to capture such complexities, intersectionality is considered essential to produce more effective and cost-efficient policies [35–39].

### COVID-19 and intersectionality: immediate aftermath to recovery phases

Initial responses to the inequitable impacts of the COVID-19 outbreak largely focused on the gendered impacts of the pandemic [40–43]^1^ but in very short order, a number of public statements, briefs and reports emerged that pointed to the importance of using a intersectional approach to understand varied risks of exposure and impacts in relation to different groups, especially those who experience intersecting forms of disadvantage [2, 7, 44–47]. As 2020 progressed, more guidance was published, including the *UN Research Roadmap for COVID-19 Recovery* [46] which called for intersectional analysis to ensure better evidence for equity and rights-promoting recovery efforts. At the same time, expert groups such as Scotland’s Expert Reference Group on COVID-19, recommended policy responses that explicitly attend to the intersections of vulnerability including socioeconomic status, migrant status, ethnicity and race [22].

**Fig. 1:**
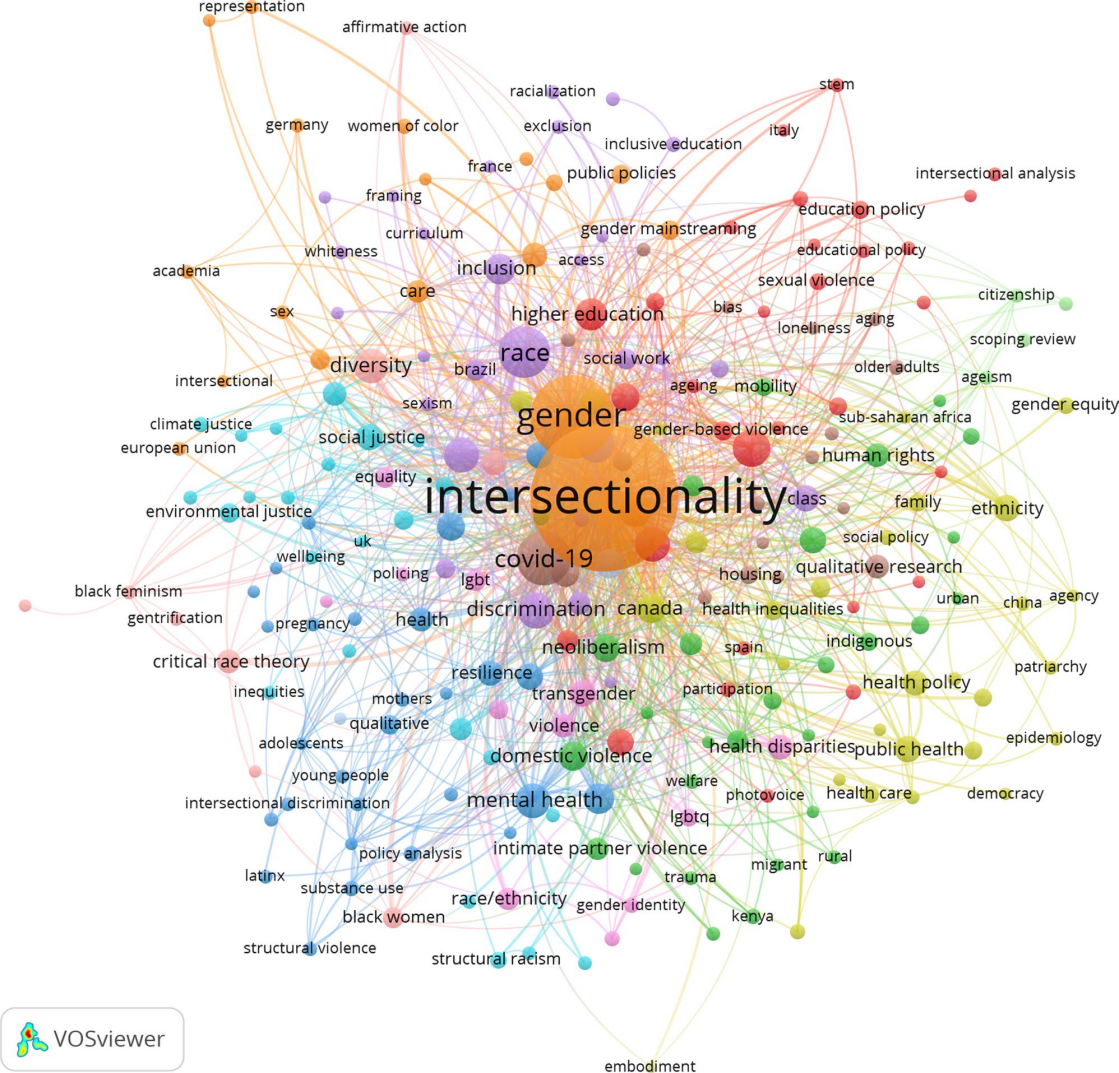
A co-occurrence analysis of author keywords that shows the joint use of keywords amongst the authors from the retrieved literature. Keywords that occurred less than five times were excluded. Created with VOSviewer

Indeed, at the height of the pandemic, there was an explosion of literature discussing the relevance of intersectionality. A review published in Spring 2021, for example, observed that in the preceding twelve months, academic and grey literature supporting intersectional analysis of and responses to the pandemic had increased significantly, covering a range of issues including: data collection and disaggregation, the need for contextual analysis, service planning and delivery, and representation in decision-making and leadership [48]. This increase in attention to intersectionality is illustrated by Fig. 1 below, showing keywords from a bibliometric analysis we conducted (1,698 articles concerning intersectionality and policy published between Jan. 2017 and May 2023), in which COVID-19 figures prominently as the keyword used the most frequently following gender.

As a shift occurred to more post-pandemic recovery efforts (late 2021 and onwards), intersectionality continued to figure prominently. The “intersectional” nature of COVID-19 and recovery was increasingly mentioned in high-level strategic documents and evidence briefs [49, 50] and multi-jurisdictional evaluations of COVID-19 impacts across diverse groups were commissioned in the pandemic’s aftermath [51–53]. Though the focus of these and most other high-level initiatives was on individual-level factors (e.g., how categories like gender, age, and geography shaped vulnerability to COVID-19) with limited to no attention to structural inequities or power, some exceptions should be noted. For example, a report documenting an interagency and expert consultation on addressing structural, racial, and ethnic based discrimination in COVID-19 recovery plans emphasizes the centrality of using intersectional analysis to “comprehensively address the multiple and overlapping structural drivers of inequality” [54, p.9]. And, UN Women’s visionary report *Beyond COVID-19: A Feminist Plan for Sustainability and Social Justice*, highlights the need for an intersectional approach that moves beyond describing compounding disadvantages that individuals or groups experience “rather than looking at the mechanisms of inequity” [55, p.8]. Similarly, UN Research Institute for Social Development’s flagship report *Crises of Inequality* argues for a new eco-social contract in the wake of the pandemic that explicitly targets inequitable power dynamics and promotes social justice via an intersectional approach [56].

Beyond these high-level calls for using intersectionality, a growing mass of researchers and community practitioners have been working to integrate it into post-pandemic efforts, across topics including gender equality, mental health, social support networks, the healthcare workforce, health care provision, access to peace and security, urban vulnerabilities, and long COVID [17, 48, 52, 56–65]. Such work is pressing, given that research to date has not sufficiently captured intersecting inequalities related to the pandemic, and that policy responses have failed to account for inequalities, often replicating and reinforcing them. Given the recognized need to better take up intersectionality as “our post-COVID legacy” [66]and the “great human cost” of not doing this [19], it is important to assess and address where the strengths and gaps lie in intersectional guidance within and beyond the pandemic.

## Methods

The literature reviewed for this article derives from a larger scoping review of documents intended to guide or facilitate the application or operationalization of intersectionality. The review drew upon the methodological approach of Arksey & O’Malley (2005) and further advanced by Levac et al. (2010), which is rigorous and well suited to capture a comprehensive range of peer reviewed and grey literature in research and policy [67–69]. We also drew upon current expert-informed protocol and a criteria checklist for conducting scoping reviews [70, 71]. This scoping review is part of a larger ongoing program of research which examines the development of intersectionality guidance published to date, as well as conducting the aforementioned bibliometric analysis of recent academic literature (2017–2023) discussing and applying intersectionality to policy.^2^ The scoping review findings also inform the first ever publicly available “living” inventory of intersectionality-informed guidance (modelled after Pauly et al. 2016; IIRP, forthcoming) [72, 73].

Our overarching research questions for the scoping review were as follows:

- What guidance (e.g., guides, tools, and/or frameworks) for applying or integrating intersectionality in policy-relevant contexts are there?
- How is intersectionality being recommended to be applied and actually applied by policymakers and practitioners?
- What are the major priorities and foci (e.g., goals, principles, settings, populations, etc.) as revealed in the guidance? Which priorities/foci seem to be overlooked or absent?

In order to identify guidance that applies/integrates intersectionality, we employed a search strategy focused on capturing guidance with the terms “intersectionality” or “intersectional” in the title. Our search consisted of the following search string:

$$\begin{aligned}I&ntersectional*\;AND\;(guide\;OR\;guidance\;OR\;framework\;OR\;tool*\cr&\;OR\;manual\;OR\;primer\;OR\;checklist\;OR\;lens)\;AND\;(policy\;OR\;policies)\end{aligned}$$

Searches were conducted to capture all potential guidance in Google, Google organisation search, EBSCO Academic Search, Web of Science, Scopus, IGO search and Policy Commons. Members of our expert research project team also signposted to guidance, and key references cited in collected guidance were scanned for potential inclusion. Google as a search engine enabled location of guidance in the public domain that aligned with search criteria. Including resources from the team also ensured that guidance no longer publicly available was included, and that no key guidance was missed. Academic search engines were employed to locate guidance that has been produced (and open access) but may not yet be published as separate “standalone” guidance outside of academic journals.

The collected guidance was screened thoroughly against the following inclusion criteria:

- Published any date up to end of 2023
- English language
- A standalone tool OR guide OR framework OR manual OR primer OR checklist OR lens for applying or integrating intersectionality
- Policy-relevant (mentions or focuses on policy or policies)

All included guidance (*n* = 95) was analysed against criteria of key tenets of intersectionality informed by Larson et al., Hankivsky et al., Ghasemi et al., and Pauly to determine, for example, intersectionality precision and potential misuse, as well as to identify where improvements should be made [37, 73–75]. Our guidance analysis tool is included in Additional File 1. It must be noted that throughout the review, much of the guidance found used the language of intersectionality (particularly in the ‘background’ sections describing inequities) but was not specifically focused on *operationalizing intersectional analysis/approaches* (such guidance was excluded from analysis). Moreover, the review excluded numerous blogs, articles, online seminars, videos, evidence reviews and research reports if they did not introduce what could be considered intersectionality or intersectionality-informed guidance. Documents in which user-friendly guidance and recommendations for operationalizing intersectionality consisted of at least 25% of the content were included.

Notably, of the 95 guidance documents which met the inclusion criteria for analysis, only 5 are specifically focused on COVID-19. It is important to note however that more than a third (37%) of all of the guidance published in 2020 and onwards mention COVID in the background or introduction. Similarly, there were many more guidance documents and websites found during the scoping review process that were focused on COVID-19 and mentioned intersectionality or intersecting factors without providing guidance for its application (thus were excluded).

This paper focuses on a total of seven intersectionality-informed guidance documents (herein termed guidances) that were developed specifically in relation to COVID-19, detailed below. Six of these were found in the original scoping review process, of which five met all of our original inclusion criteria (Molenaar 2021) [76] did not meet the criteria of having a minimum component of 25% focused on guidance/recommendations). We included a seventh additional document not found through our scoping review search methods, but rather a supplementary search using our search string and the additional term “COVID” (Wangarĩ Kamunge 2021) [77]. These latter two documents have been included to inform a more robust and current analysis of COVID-focused guidance documents for operationalizing intersectionality.

### Results - COVID-19 intersectionality frameworks

As aforementioned, our findings indicate limited COVID-19-oriented guidance as compared with what might be expected, given overwhelming increasing discussion of COVID-19 in relation to intersectionality [7, 21, 66, 78] as well as the increased production of intersectionality guidance since the outbreak.^3^ In the following tables, we summarize essential content of this guidance (presented chronologically), with a focus on the stated purpose and recommendations.

**Table:** 

Guidance 1
Hankivsky, Olena, and Anuj Kapilashrami. “Beyond Sex and Gender Analysis: An Intersectional View of the COVID-19 Pandemic Outbreak and Response.” University of London, 2020. https://mspgh.unimelb.edu.au/__data/assets/pdf_file/0011/3334889/Policy-brief_v3.pdf.
This policy brief produced near the onset of the pandemic provides high-level guidance on understanding and addressing intersectionality in responses to the COVID-19 pandemic. It describes the limitations of focusing on sex or gender differences alone in comparison to an intersectional approach, lays out the definition and value-added of intersectionality, provides examples of how social locations such as age/health status, migration status, and geographic location interact to shape COVID-19 risks, and shows examples of how policy mitigation measures and pandemic impacts differentially effect diverse groups. It then proposes eight “recommended actions” for intersectionality-informed policy and decision making around COVID-19, including:
• Move beyond sex disaggregated data using a gender perspective – integrate social factors such as gender norms, age, disability, etc. into data collection and analysis
• Collect diverse data – generate data from diverse sources, prioritize research partnerships and multi-method case studies, and include the voices of those affected
• Contextualize data – place reports of pandemic effects in broader social and structural contexts
• Undertake an intersectionality analysis of national and global responses – move beyond prioritizing gender towards human rights and intersectionality-relevant factors
• Broaden bailout and stimulus packages to prioritise those most at risk - including the extension of amnesty provisions to migrants and special protection measures
• Make policy responses cross-sectoral – coordinate across diverse sectors and tailor communications to specific contexts
• Move beyond a deficit model – decision makers should emphasize resilience, agency, and strength and prioritize engagement among affected populations
• Commit to leadership diversity – ensure policy makers and leaders are diverse, including across gender, geography, ethnicity, etc.

**Table:** 

Guidance 2
**Conducting COVID-19 Rapid Assessments in Jordan: Integrating Gender and Intersectionality [Internet]. UN Women Jordan; 2020. Available from:**https://jordan.unwomen.org/sites/default/files/Field%20Office%20Jordan/Images/publications/2020/May/COVID-19%20Guidance%20note_%20Gender%20rapid%20impact%20assessments.pdf
This short guidance note developed by UN Women Jordan in the early months of the outbreak aims to guide a rapid gender and intersectionality informed analysis in the context of COVID-19 in Jordan. Informed by progressive intersectionality-aligned research, policies, frameworks and tools (listed in a comprehensive annex), it begins by discussing the value added and necessity of an intersectionality approach that considers intersections of gender with other dimensions of diversity and inequity in the context of understanding and mitigating the impacts of COVID-19. It provides four steps, each accompanied by practical guiding questions, to help provide full understanding of issues and experiences of diverse groups and inform action to leave no one behind:
• Step 1 - baseline data collection – includes creating an inventory of information of what was known before the outbreak, including qualitative and quantitative data relevant to intersecting forms of inequality
• Step 2 - collect COVID-19 related data – includes addressing data gaps needed to capture how the differential impacts of the pandemic, mitigation measures, social protection measures and financial aids
• Step 3 – analyze collected information - includes comparing data collected at earlier stages to understand how measures impact or fail to impact different groups.
• Step 4 - make and share practical recommendations – includes developing accessible recommendations that prioritize actions that address inequalities, and sharing both analysis and recommendations with diverse stakeholders.

**Table:** 

Guidance 3
**D’Ignazio C, F. Klein L. Seven intersectional feminist principles for equitable and actionable COVID-19 data. Big Data & Society [Internet]. 2020 Jul.;7(2):205395172094254. Available from:**http://journals.sagepub.com/doi/10.1177/2053951720942544
This commentary published in the summer of 2020 is aimed at researchers and activists working with data and within data science. It offers seven intersectional feminist principles for equitable and actionable COVID-19 data, drawing from the authors’ prior work on data feminism. For each principle, they provide examples – mostly from the U.S. - focused on identifying and addressing power imbalances related to COVID-19 impacts and responses. Potential ways to take up each principle in relation to data feminism are provided, including questions to ask, potential methods, and sources of knowledge. The principles include:
• Examine power – begin by analyzing how power operates
• Challenge power – commit to challenging unequal power structures
• Elevate emotion and embodiment – value multiple forms of knowledge
• Rethink binaries and hierarchies – challenge the gender binary and other classification systems
• Embrace pluralism – synthesize multiple perspectives, prioritizing local, Indigenous and experiential ways of knowing
• Consider context – acknowledge data is not neutral or objective but a product of unequal social relations
• Make labor visible – recognize and value the work of all involved in data science.

**Table:** 

Guidance 4
**Gender and Intersectionality in Rapid Assessments [Internet]. UN Women Jordan; 2020. Available from:**https://jordan.unwomen.org/sites/default/files/Field%20Office%20Jordan/Images/publications/2020/September/Gender%20%20Intersectionality%20in%20Rapid%20AssessmentsUNW.pdf
This guidance note authored by UN Women Jordan and published in the fall of 2020, aims to assist policy and programme actors to conduct rapid assessments that are responsive to both gender and intersectionality. After articulating the importance of considering gender and intersectionality in the context of COVID-19 impacts and mitigation measures, it provides guidance on integrating gender and intersectionality into the design of rapid assessment questionnaires and surveys. Guidance is provided across three phases of rapid assessments: developing assessment surveys, implementing them, and analysis of findings and resulting recommendations. Sample questions and key resources are cited throughout the document. Examples of content for each phase include:
• 1) Survey development – e.g., when deciding on purpose, consider whether subgroups of people will be targeted and why?; ensure that questions are informed by technical experts and affected population groups; ideally disaggregate data my multiple diversity factors; do no harm in data collection;
• 2) Implementation – e.g., be transparent about who is conducting assessment and purpose; ensure familiarity with gender and intersectionality; consider potential interviewer bias;
• 3) Analysis/Evidence generation – e.g., ensure analysis driven by experts across sector of concern, survey research, and gender and intersectionality; articulate differential outcomes between subgroups, sources of strength and resilience in affected groups, and how proposed actions will impact diverse groups.

**Table:** 

Guidance 5
**Molenaar J. Using an intersectional lens to understand the unequal impact of the COVID-19 pandemic. [Internet]. 2021 Sep. (Bi-monthly report 5). Report No.: COVINFORM H2020 Project No. 101,016,247. Available from:**https://www.covinform.eu/wp-content/uploads/sites/39/2021/09/COVINFORM-Brochure-A4-Bi-Monthly-Report-5.pdf
This high-level report published by the COVINFORM Consortium project (partnership of primarily EU and UK countries), aims to show the value of intersectionality in understanding how structural inequalities shape COVID-19 risk, and how mitigation measures interact with these inequalities. It includes examples of how responses to the crisis, such as mobility restrictions and school closures, are differentially experienced depending on people’s positions in society.
Strictly speaking, it is not a framework for applying intersectionality according to our inclusion criteria since the majority of its content presents intersectionality-informed research rather than guidance, thusly *not meeting our inclusion criterion* of providing guidance as at least 25% of content. However, we include it here because it lists four important recommendations on promising practices for including an intersectional lens in responding to the COVID-19 pandemic and similar crises. These are:
• collect diverse data that is disaggregated by various social characteristics – from multiple sources, using multiple methodologies, and contextualized
• coordinate cross sectoral responses across sectors like education, housing, and social protection
• place intersectional perspectives at “the heart of welfare states” to address both causes and symptoms of inequalities
• support and promote leaders from a range of communities and local organizations using resilience and asset-based approaches as well as participatory engagement mechanisms

**Table:** 

Guidance 6
**Wangarĩ Kamunge B. Which inequalities should we focus on in evaluating health policy before, during, and following COVID-19? Ethical Framework. Bristol, UK: UK Pandemic Ethics Accelerator; December 2021. Available from:**https://ukpandemicethics.org/wp-content/uploads/2021/12/Intersectionality-Framework.pdf.
This framework provides high level guidance on how to conduct an intersectionality analysis of policy. It first provides a summary of inequalities in the context of the pandemic and some key factors shaping these, and an overview of intersectionality and how it can facilitate understandings of such inequalities. It then lays out a “framework of questions” along with justification for these questions, as a starting point for intersectionality analysis. This framework was found *in a supplementary search outside of our scoping review*.
Suggested questions include:
• Whose care is seen as the type of care that can be left waiting? (reflect not only on maximising health benefits, but also on marginalizing impacts of decision making)
• Who gets to breathe during a pandemic? (consider differential material and structural privilege and disadvantage in relation to impacts of COVID-19 and response measures)
• Whose voice is (not) being heard? (recognize decision making as value-laden and ensure voices of different disadvantaged groups are heard and new knowledges created)
• How did we get here? (draw from past lessons learned to see current inequalities as a “norm made worse”, and make stronger connections between interlocking structures producing this norm)

**Table:** 

Guidance 7
**Haaris Tiwana M, Mũrage A, Smith J. Applying intersectional approaches to pandemic preparedness and response. [Internet]. Vancouver, BC, Canada: Pacific Institute on Pathogens, Pandemics, and Society, Simon Fraser University; 2023 Aug. Available from:**https://pipps.cdn.prismic.io/pipps/dcf401eb-70dd-4500-b99a-1ad10a982f99_Intersectionality+Brief+Aug+2023.pdf
This research brief published in the summer of 2023 provides high level guidance on how intersectional approaches might be applied to pandemic preparedness and response in order to ensure health and human rights for all. It provides an overview of intersectionality including five key tenets, and how these tenets can be operationalized using evidence and lessons learned from COVID-19 to illustrate.
Intersectional tenets recommended include:
• emphasize relationality of social identities – move beyond single identity groups, expand data collection and apply intersectional data analysis
• focus on how oppressive systems intersect – including how systems and structures of privilege and oppression shape risk and mitigation response effects
• pay attention to social and historical contexts – beyond one size fits all responses, including intersectionality and cultural-safety informed training
• position oneself within intersectionality work – require self-reflexivity including historical harms of public health practices, and openness to alternative approaches
• examine systems of power in which intersectional work is done – include opportunities for meaningful engagement and inclusion of priority populations in decision making

## Discussion

The findings of our review show that COVID-19 provided a watershed moment for intersectionality, including increased recognition that the status quo was insufficient to address inequity, and that an intersectional approach is required. However, despite the increased rhetoric around intersectionality, only seven guidances were found via the review and a supplementary search that provide concrete direction as to its implementation in relation to the pandemic. This is also surprising in the context of our broader scoping review of intersectionality guidance produced up to the end of 2023, which found that almost three quarters of all guidance (64 of 95 total) were produced in 2020 and onwards [29].

### Defining and grounding intersectionality

In terms of positioning themselves in reference to critical intersectionality theory and frameworks, five of the guidances can be considered to draw on the Intersectionality-Based Policy Analysis (IBPA) Framework (Hankivsky et al. 2012) - the first of its kind guidance on applying intersectionality across policy-relevant fields and disciplines [79]. Specifically, three guidances (Hankivsky & Kapilashrami 2020 [80] UN Women 2020a [81]; Molenaar 2021 [76]) explicitly discuss IBPA and both Molenaar (2021) [76] and Haaris Tiwana et al. (2023) [82]cite and build upon recommendations made in Hankivsky and Kapilashrami (2020) [80]. Similarly, the second guidance published by UN Women (2020b) [83] cites and refer to their preceding guidance (2020a) [81] which draws on IBPA as a key resource on intersectionality. This reflects the increasing international recognition and use of the IBPA framework – which contains guiding principles and questions to operationalize intersectionality, along with case examples demonstrating its application - as the most ambitious effort to date in intersectional public policy guidance [75].^4^ With respect to the remaining guidances, D’Ignazio and Klein (2020) [84] and Wangarĩ Kamunge (2021) [77] do not reference other frameworks, but both draw on an “intersectional model of power” – the “matrix of domination” proposed by Patrica Hill Collins [30]. And overall, to different extents, all of the guidances importantly cite references that capture the critical theoretical and social justice-oriented work of Black feminist activists and scholars within their discussions of intersectionality.

Given that each document grounds itself in foundational literature on intersectionality, it is not then surprising that each presents robust and accurate definitions of what intersectionality is, including how it improves upon the status quo in equity-oriented policy work, within and beyond the context of COVID-19. For example, all of the documents define intersectionality in ways that do not position one particular category or inequality as being a priori more important than others. In the guidances where gender analysis is foregrounded in background, the value added of moving beyond gender to intersectional analysis is emphasized and gender is situated as inextricable from other axes of diversity and structure and processes of power (Hankivsky & Kapilashrami 2020 [80]; UN Women, 2020a [81], 2020b [83]). For example, UN Women (2020b) states: “Intersectionality directs attention to the fact that gender interacts with not only age and disability but multiple factors such as nationality, ethnicity, underlying health conditions, geography, socio-economic status, and migration or refugee status [83]. It highlights that the impacts of COVID-19 cannot be understood without attention to the broader context – from political systems, laws, and policies to power structures such as patriarchy, racism, ageism, nationalism, and xenophobia” [83 p.1].

### Taking up intersectionality’s tenets

Promisingly, across all of the analyzed documents, intersectionality is taken up in ways that uphold its underlying tenets. First, a central tenet of intersectionality, critical self-reflection (reflexivity), is explicitly emphasized as central to intersectional analysis in D’Ignazio and Klein (2020) [84] as well as Haaris Tiwana et al. (2023) [82]. In addition, a focus on **reflexivity** – though not explicitly discussed - is arguably reflected in the four frameworks that are informed by IBPA (Kapilashrami & Hankivksky 2020 [80], UN Women 2020a [81], 2020b [83]; Molenaar 2021 [76]) given that IBPA positions reflexivity as a key starting point and principle in any analysis. And, Wangari Kamunge (2021) problematize constructions of decision making as impartial, calling for policy processes that lay bare the “value laden nature of decision making” and encouraging a “humble posture” via reflection on whose voice is and is not being heard [77 p. 6–7]. Critical reflection on policy actor positionality, including potential biases, values, and power – though central to intersectional analysis – is often missing from policy-relevant frameworks espousing to be equity-informed or even “intersectional”, as our wider scoping review shows (Christoffersen, Hunting and Hankivsky 2025) [29].

Second, all of the guidances emphasize the **intersecting and dynamic nature of multiple factors** shaping inequity. Specifically, they do not explicitly focus on any single or assumedly independent list of factors in analyzing inequities. This is critical to progress past the tendency of equity-oriented guidance to begin with the assumption that categories such as gender are most important, or to prioritize a focus on specific factors such as the “gender, race, class” triad alone. Such an open-ended approach is particularly important in the context of pandemic-related inequities, where factors such as urbanization, digitalization, housing, and migration were all shown to intersect with social locations such as gender and socioeconomic stats to shape exposure, vulnerability, and recovery outcomes. An intersectional approach demands attention to such context-specific and emergent dimensions of inequity, which may not be captured by pre-determined category lists but which are essential to understanding how pandemics produce and deepen disparities. However, at times within the guidances, a finite list of factors of importance is emphasized, both in Molenaar (2021) [76] - e.g. gender, age, socio-economic status, disability, family composition, race/ethnicity, and migrant background - and D’Ignazio and Klein (2020) [84] – e.g., gender, race and class. These statements are not consistent with intersectionality, which recognizes that relevant factors are dynamic across time and place, and that determination of relevant factors in any analysis should remain open ended [33]. Importantly, however, across all documents, categories of analysis are positioned as not independent of each other or additive, but rather mutually constructed, interdependent and co-constituted. Also important is that they all discuss the relevance of multi-level factors in shaping pandemic experiences, including rural or urban location, insecure or higher-exposure employment, globalization, cultural acceptability, and access to digital resources and interventions.

Third, **power dynamics** – including those operating at institutional, political and structural levels – are mentioned at multiple points across the documents as central to shaping inequities. In addition, to different extents, all documents also acknowledge the need to consider how such dynamics confer privilege and advantage to individuals and groups. This “messiness” of power relations that intersectionality espouses – namely that people experience both advantage and disadvantage across time and space – is a fact often absent in policy frameworks which focus on addressing vulnerability and risk as static phenomena among certain population groups. This can reduce and stereotype certain populations as inherently at risk or vulnerable in relation to policy problems, overlooking shifting power dynamics and how these dynamics confer advantage to certain groups. Countering this trend, D’Iganazio and Klein (2020) describe power as “the current configuration of structural privilege and structural oppression in which some groups experience unearned advantages—because systems have been designed by people like them and work for people them” [84, p.2]. And, Harris Tiwana et al. (2023) emphasize the historical and context-specific nature of policies in times of crises: “crisis response has been used to justify colonialism, the forced treatment and evictions of racialized populations, and more. Responses often aim to protect the privileged at the expense of those most at risk due to structural inequities” [82, p. 5].

### Action towards social justice

All of the frameworks recognize that the onus for action on the inequities relating to COVID-19 does not only lie on particular groups in particular sectors (e.g., health policy actors). Sectors and systems including social welfare, health systems, employment, food security, education, etc. are all discussed as important in both shaping and responding to such inequities, prior to each document providing key recommendations for policy actors. Yet, only four of the seven guidances are more explicit in recommending cross-sectoral and/or cross-organizational collaboration, partnerships, and knowledge exchange [76, 80, 81, 83]. This may reflect a common disjuncture in policy guidance where policy problems can be well described, but concrete action for policy actors to take are not made explicit. Hankivsky and Kapilashrami (2020) for example, emphasize the importance of cross sectoral and transnational policy responses, as well as engagement mechanisms, particularly for those “who despite greater risks may possess strong political and social awareness and collective organizations and coalition building experience and insights” [80]. Such engagement as a core component of operationalizing intersectionality, is essential to ensure that policy actions promote the well-being of all.

Importantly, all of the guidances recommend the prioritization of meaningful engagement of populations most effected by policy, particularly those experiencing multiple forms of marginalization who have been historically excluded from decision making processes. For example, with respect to key stakeholder representation, UN Women’s (2020b) guidance asks: “Are community groups, specialized NGOs and voices of marginalized segments of communities, involved in an iterative process of questionnaire design, including the identification of key issues and problems to be evaluated, plans for implementation and data analysis and recommendations regarding policy solutions?” [83, p. 2]. Centring the voices and knowledges of such groups as experts of their own histories, experiences and needs – from the outset of any policy process - is critical to buttress the trend of watering down the transformative potential of intersectionality. Wangari Kaumge’s (2021) guidance underscores the importance of this: “Asking whose voice is (not) being heard encourages a humble posture from which new knowledges can be created, and inequalities tackled. The question also engenders a critical posture on the nature of public discourse. More needs to be done to ensure that the voices of different disadvantaged groups are heard.” [77, p. 7].

### Strengthening intersectional guidance

Overall, this analysis of COVID-19 guidances showed that they move beyond the recognized limitations in guidance purporting to be intersectional. However, as compared with our wider scoping review, the guidance developed, albeit limited, is on the whole of a higher quality than other guidance on operationalising intersectionality which is not specific to Covid-19 [29].

Importantly, and like many critical intersectional scholars to date, Haaris Tiwana et al. (2023) [82] emphasize this tendency for intersectionality to be taken up in ways that lose its critical stance, and also further mention, along with D’Ignazio and Klein (2020) [84], the sometimes uncritical appropriation of intersectionality by scholars in positions of privilege, including white scholars, and the need to critically assess and address this in intersectional work. It is thus important to pay close attention to how intersectionality is interpreted and taken up to ensure its application sparks social transformation and equity to avoid its appropriation into systems and practices that reinforce relations of exclusion [85–88]. The strength in all of the guidances analyzed is their recognition of the underlying principles of intersectionality, which facilitate their critical interpretation and use.

In accordance with the larger scoping review findings, guidance author reflexivity is largely absent in the frameworks analyzed, with the exception of D’Ignazio and Klein’s (2020) footnote to more information on their positionalities within the content of the book from which the article draws [84]. This absence mirrors a trend in a recent scoping review of the operationalization of intersectional health research methods in relation to COVID-19 which found that the studies analyzed “offered little in terms of reflexivity” [89 p. 7]. Though reflection on decision maker subjectivities and biases rarely occurs in governance and policy processes which value assumedly “objective” methods, critical research and policy work has increasingly shown that public policy and population well-bring “can be improved both epistemically and ethically” via reflexive practice [89].

There also remains a recognized need for policy guidance that accounts for the context in which users are motivated or enabled to take up said guidance. Equity informed guidances often emphasize what needs to be done, but do not typically accounted for the barriers and facilitators to their use in particular policy contexts, which plays a large role in the extent to which they are taken up, and their effectiveness [90–92]. In the analyzed guidances, barriers to overcome are often implied in the recommendations, however, there is very limited focus on how to address or work within such barriers to better facilitate guidance uptake. For example, they all point to the need for better data and evidence, providing tips on how to do this (e.g., community consultation, disaggregating data, etc.) but provide limited detail on how to support this given common institutional or organizational barriers to equity-promoting and intersectional work, such as having the required expertise, the time and resources for robust evidence generation, lack of political will, etc.) [25, 92, 93]. Though this level of detail may not be provided given the need for guidance to be short and digestible, there remains a need to ensure the relevance and usability of guidance for policy actors in different environments. As intersectional scholarship continues to highlight, addressing the barriers and challenges and building capacity around taking up intersectional policy guidance – and demonstrating how this is actually being done in various jurisdictions via case examples and knowledge exchange - remains a key priority to ensure transformative policy action and change. This includes sharing knowledge around how to best get policy actors onboard with operationalizing intersectionality, providing space and time to discuss its value-added and engage in reflexive dialogue and training, leveraging existing institutional mechanisms as entry points for intersectional analysis (particularly relevant in resource-constrained settings), and working in partnership with system actors and civil society on policy change [29, 93–95].

## Limitations

Our scoping review and analysis provides an in-depth overview of intersectional guidance in relation to COVID-19, including key foci, strengths, and gaps. This analysis does not include potential guidances that do not include the term “intersectional” or “intersectionality” in the title, but a preliminary scoping review of guides to date in which intersectionality is the central focus but not in the title found none that were specifically focused on COVID-19 and met our inclusion criteria. We also recognize this analysis excludes non-English language guidances as well as COVID related guidances that consider and incorporate principles of intersectionality (e.g., social justice and human-rights approaches) but do not explicitly take an intersectional approach. The English-language restriction is a notable limitation given the global impact of the pandemic and evidence that intersectionality is increasingly being engaged with in non-Anglophone scholarship and public policy contexts, including in Latin America, francophone Africa, and South and Southeast Asia. As scholars have noted, a predominant focus on Anglophone discussions and frameworks related to intersectionality, risks obscuring context-specific non-Anglophone conceptualizations, guidances, and related policy relevant actions [29, 96, 97]. Future research and knowledge exchange initiatives should actively prioritize multilingual and cross-regional collaborations, including partnering with researchers fluent in non-English languages and with experience in non-Anglophone policy environments, to better capture a global sense of intersectionality-informed guidance and its operationalization in pandemic recovery and beyond. It is thus important that future intersectional guidance development look across such tools to find areas of synergy, forge alliances and work to promote equity in policy collaboratively.

## Conclusion

The COVID-19 pandemic broadened awareness of the ways in which inequities are intersecting and not being adequately mitigated. In this paper we have sought to explore whether this raised awareness provided a further stimulus for advancing operational guidance for intersectionality. We have presented findings concerning COVID-19 specific intersectionality guidances from our broader scoping review of operational guidance for intersectionality. We have found that while there was an explosion of literature concerning intersectionality and COVID-19 following the pandemic, and many calls for using an intersectionality perspective to better understand and respond, just seven COVID-19 specific intersectionality frameworks were developed and located in our initial and supplementary search. However, each of these present promising practices in relation to the wider field of intersectionality frameworks, especially in terms of emphasising intersectionality’s foundational tenets. Moreover, frameworks developed since the pandemic, though not pandemic specific, may have been propelled forwards by the utility and increasing popularity of intersectionality to understand inequities relating to Covid-19.

The COVID-19 pandemic is often considered over, but its individual, social and structural impacts persist, as well as groundswell to ultimately change how policy issues are conceived and addressed in ways that are responsive to complexity, and prioritize and promote equity. Yet a recent analysis of intersectionality’s application in the context of the pandemic highlights that policy responses have lacked a robust intersectionality-informed approach, and that “new thinking on how public policies can better recognize and respond to the intersectional nature of experiences of inequality” exposed by COVID-19 are necessary [66, p 1206]. Our findings underscore what has been recently highlighted by intersectional practitioners elsewhere [29, 94, 98, 99]: there is a continued need to critically evaluate, discuss, and draw on lessons learned in the field of intersectionality guidance and guidance operationalization, from diverse disciplines and sectors, in order to harness the potential impact of an intersectionality-informed approach in ways that are accurate and transformative.

## Electronic supplementary material

Below is the link to the electronic supplementary material.

Supplementary material 1

## Data Availability

The datas used and/or analyzed for this manuscript are available from the corresponding author on reasonable request.

## Footnotes

1 For a detailed list of the many reports, guidelines, statements, research, news, and commentaries focused on COVID-19 and gender see for example: https://genderandcovid-19.org/resources-page/ and https://data2x.org/resource-center/gender-and-data-resources-related-to-covid-19/.

2 Canadian Institute of Health Research Project Grant (PJT-180461, 2022–2026) on which Co-Author Hankivsky is the Primary Applicant.

3 Our recent scoping review of intersectionality guidance (published to December 2023) found that 64 of the 95 total guidances to date (~67%) were published between 2020–2023 [28].

4 Since its publication in 2012, IBPA has been taken up in over 30 countries and across various disciplines, sectors, and health and social topics, including COVID-19 [23, 80, 94, 100–104]. A revised version of this framework – IBPA 2.0 – is forthcoming in 2026, accompanied by a series of linked micro-primers across policy relevant foci (e.g., budgets, KPIs, quantitative research), informed by the findings of a 4.5 year research project gauging input from intersectional researchers, policy actors, and practitioners globally on how the framework can be made more context relevant, user friendly and impactful. For more, see http://www.intersectionalityinstitute.ca.
