# Supplementary material for: Taking stock of intersectionality-informed policy guidance for post-pandemic recovery: a scoping review
Source: Int J Equity Health. 2026 May 23;25:172. doi: 10.1186/s12939-026-02853-8 (PMC13397774; doi:10.1186/s12939-026-02853-8)
Supplement: Supplementary file 1 — Supplementary material 1 [file 12939_2026_2853_MOESM1_ESM.docx]

# Additional File 1: Intersectionality Guidance Analysis Tool

1. Title:
2. Reviewer:
3. Inclusion: Is a standalone tool OR guide OR framework OR strategy OR program* for applying or integrating intersectionality with intersectional* or intersectionality in title?

Yes - No

1. Is the goal or purpose of the tool clearly identified?

Yes – No – Not sure

4.a. If yes, what is its stated goal/aim/objectives? (copy/paste)

1. Who are the intended audience/users? (select all that apply)

Policy makers (specify)

Practitioners (specify)

Researchers

Community

Other

Unclear

Organisations (specify)

5.a. Specify if a more specific group of policymakers is named

5.b. Specify if a more specific group of practitioners is named

5.c. What kind of organisation

5.d. If you selected Other, please specify:

1. Originating country:
2. Is it focused on a particular population/inequality/intersection? (e.g. people with

disabilities)?

Yes – No

7.a. Which population/inequality/intersection?

1. Is it focused on a particular issue or health issue? (e.g. HIV)

Yes – No

8.a. Which issue?

1. Does it position itself with other tools and frameworks in existence?

Yes – No

9.a. Specify

1. Are references provided to support the theoretical orientation or the empirical

basis?

Yes – No – Not sure

10.a. Specify main references

1. Is intersectionality defined?

Yes – No

11.a. Copy and paste definition

1. Is intersectionality used in an additive or a mutually constitutive way?

12.a. Additive

12.b. Mutually constitutive

12.c. Not sure

12.d. Both (at different points in the document)

1. Is one inequality framed as being more important than others?

Yes – No - Not sure

13.a. Specify

1. Is there focus on structural and political factors which shape inequalities?

Yes – No – Not sure

14.a. Details - to what extent?

1. Is there a focus on (check all that apply)

15.a. Vulnerability of people affected by inequities

15.b. Agency and/or resistance of people affected by inequities

15.c. Not sure/unclear

1. Is author/organisation reflexivity discussed relating to production of the

tool/guide/framework?

Yes – No – Not sure

1. How is it intended to be used? (e.g. steps involved in using it)
2. Is reflexivity a factor of the tool/guide/framework?

Yes – No – Not sure

1. Is addressing structural and political factors which shape inequalities a factor of the

tool/guide/framework?

Yes – No – Not sure

1. Are the contexts (including barriers/facilitators) of using the tool discussed or

considered?

Yes – No – Not sure

20.a. Barriers and/or facilitators

1. Is there a step in the tool that engages or calls for participation of the community or people affected by inequities?

Yes – No – Not sure

1. Are examples provided where the tool been applied to a policy, program, etc? (e.g.

case studies)

Yes – No – Not sure

22.a. More information on examples or case studies

22.b. If the tool has been applied, has the tool been formally evaluated for effectiveness?

22.b.i. Details of evauation

1. Any other comments
